# The Influence of Environmental Conditions on Intake Behavior and Activity by Feedlot Steers Fed Corn or Barley-Based Diets

**DOI:** 10.3390/ani11051261

**Published:** 2021-04-27

**Authors:** Hannah M. DelCurto-Wyffels, Julia M. Dafoe, Cory T. Parsons, Darrin L. Boss, Timothy DelCurto, Samuel A. Wyffels, Megan L. Van Emon, Janice G. P. Bowman

**Affiliations:** 1 Department of Animal and Range Sciences, Montana State University, Bozeman, MT 59717, USA; timothy.delcurto@montana.edu (T.D.); megan.vanemon@montana.edu (M.L.V.E.); jbowman@montana.edu (J.G.P.B.); 2 Northern Agricultural Research Center, Montana State University, Havre, MT 59501, USA; jdafoe@montana.edu (J.M.D.); Cory.Parsons2@chsinc.com (C.T.P.); dboss@montana.edu (D.L.B.); samwyffels@montana.edu (S.A.W.)

**Keywords:** barley, behavior, corn, environment, intake, steer

## Abstract

**Simple Summary:**

Cattle wintered at northern latitudes are often exposed to periods of severe cold. Cattle likely alter feed intake and behavior to combat environmental challenges. This study evaluated the influence of diet and environmental changes on intake behavior and activity (lying time) of feedlot steers. Short-term temperature changes impacted both beef feedlot cattle intake behavior and activity. The steers’ diet, whether they were fed corn or barley, interacted with short term environmental changes to influence animal feeding behavior, but diet had limited impact on cattle lying behavior. Lying behavior was influenced by short-term temperature changes in which cattle spent more time lying down on relatively cold days. Overall, environmental shifts and cold temperature conditions could result in greater energetic needs and ultimately impact feedlot steer intake behavior and activity. By providing information related to beef cattle feedlot behavior, we can more effectively manage cattle feeding systems at northern latitudes to improve feed efficiency.

**Abstract:**

This study evaluated the influence of diet and environmental conditions on intake behavior and activity of feedlot steers. Feedlot rations used were comprised of a main concentrate: (1) corn or (2) barley. A GrowSafe system measured individual animal intake and behavior and HOBO accelerometers measured steer standing time. An Onset weather station collected on site weather data. Steer daily intake displayed a diet by temperature class interaction (*p* ≤ 0.05). Relative temperature change had no effect on variation in intake (*p* = 0.60); however, diet influenced variation of intake (*p* < 0.01), where corn-fed steers had a greater coefficient of variation (CV) than barley-fed steers (21.89 ± 1.46 vs. 18.72 ± 1.46%). Time spent eating (min d^−1^) and eating rate (g min^−1^) both displayed a diet by temperature class interaction (*p* ≤ 0.05). Diet did not affect steer lying activity (*p* ≥ 0.12), however, time spent lying (min d^−1^) and frequency of lying bouts (bouts d^−1^) increased on relatively cold days while the duration of lying bouts (min bout^−1^; *p* < 0.01) decreased. Short-term environmental temperature changes interacted with diet influencing feedlot beef cattle intake behavior; however, they did not interact with basal diet in respect to steer activity.

## 1. Introduction

Environmental conditions and their impact on beef cattle production have long been recognized [1,2,3], and seasonal variations of climatic conditions have been documented to impact feedlot cattle performance [4,5]. Cattle wintered at northern latitudes are often exposed to periods of severe cold, which increase energy expenditure to maintain homeothermy [6,7]. Thus, during periods of cold stress, animal behavior is altered [8], and feed consumption will often increase with a decrease in average daily gain (ADG), resulting in an overall decline in feed efficiency [9]. Research conducted over seven years at the University of Saskatchewan feedlot reported that from December to February (mean monthly temperature −17 °C), ADG decreased 30% compared to the remainder of the year [5]. Regression equations relating mean air temperatures and climatic stress to relative performance indicate that 40 to 60% of the seasonal variation in feedlot performance can be accounted for by climatic variables [9]. Inherently cattle likely alter their behavior to combat these environmental challenges.

Feeding high-quality and energy dense feed sources are essential to meet energy demands for feedlot cattle at northern latitudes. Corn has traditionally been the most popular grain source in the U.S. [10]; however, barley is commonly fed to beef cattle in the northern U.S. and Canada due to its adaptation to the environmental conditions at northern latitudes [11]. Although the National Research Council reports lower energy values for barley than corn [12], work by Bowman et al. [13] suggests that barley and corn often have similar net energy values. However, the utilization of these different feedstuffs has been shown to yield differences in performance [14,15,16] and digestive utilization in feedlot steers [17,18].

Feeding behavior and activity are frequently monitored to evaluate cattle well-being and health status [19,20]. However, limited work has been conducted in regard to cattle behavioral changes, both feeding and activity behavior, when fed differing basal diets and under variable environmental conditions. Feed intake and subsequent utilization by the animal involve complex biological processes, as well as interactions with environmental factors [21]. The influence of environmental conditions and differing finishing diets on cattle feedlot behavior remains to be fully defined. Therefore, the objectives of this study were to evaluate the effects of changing environmental conditions at northern latitudes on feeding behavior and activity of steers fed corn or barley-based finishing diets. We hypothesized that steer behavior is affected by both diet and environmental conditions.

## 2. Materials and Methods

All protocols and procedures were approved by the Agriculture Animal Care and Use Committee of Montana State University (#2016-AA26). This study was conducted at the Northern Agricultural Research Center in Havre, Montana (48.5500° N, 109.6841° W). All animals were provided by the Montana Agricultural Experiment Station.

Angus-based yearling steer calves were fed in a feedlot trial from February to June (105 days) for two consecutive years (year 1, 427.3 ± 3.7 kg, n = 48; year 2, 406.8 ± 3.4 kg; n = 47). All steers were implanted with a Synovex One Feedlot Implant (Zoetis, Parsippany-Troy Hills, NJ, USA) at the initiation of the study. In addition, steers were stratified by body weight (BW) and assigned to one of two primary basal grain dietary treatments: (1) Number 2 feed corn or (2) Hockett barley. Hockett barley is a two-rowed dry-land malting variety of barley, that is often fed to livestock when malting parameters are not met [22]. Both barley and corn were dry-rolled, and diets contained 80% grain, 12% barley straw, 3% canola oil, and 5% supplement averaging 10.28% crude protein and 0.24 Mcal kg^−1^ net energy gain. Supplements consisted of vitamin/mineral packages for feedlot steers and protein sources, including wheat middlings and canola meal. Steers were acclimated to their respective diet for 14-days prior to the start of the data collection period. Steers were fed their respective diets once daily at 08:00 and managed to allow for maximum individual intake without excessive feed refusals. Bunks were read at 12:00 daily, and when clean for two consecutive days, rations were increased by 0.23 kg per head. Feed refusals were removed weekly. All animals had ad libitum access to water throughout the study period.

Steers were fitted with an electronic identification ear tag and allotted to one of 12 pens (6 pens per treatment; 4 steers per pen) measuring 5 × 11 m in size. A total of 24 GrowSafe (GrowSafe Systems Ltd., Airdrie, AB, Canada) electronic feed bunks (12 per treatment; 2 per pen) were used in this study. Steers were adapted to the GrowSafe system for 14-days prior to the start of the study. Each GrowSafe feed bunk was equipped with an antenna to detect animal presence and neck bars that allowed for only one animal to enter the feed bunk. Load cells then measured feed disappearance. Individual animal intake was recorded daily via wireless transfer to a data-acquisition computer. The GrowSafe system monitored unaccounted feed disappearance daily, and when over 5% of the feed disappearance was unaccounted for, the GrowSafe system automatically deemed the 24-h period as failed. In our study, 8.54% of the dry matter intake data failed in year 1, and 10.92% failed in year 2, with an average fail rate of 9.73% across both years. Prior research validating the use of GrowSafe has demonstrated that the accuracy of dry matter intake was not impacted when up to 30% of the data were missing [23].

Steer weights were obtained at the beginning and every 28 days throughout the study until the end of the feeding trial. Feeding behavior measurements: daily dry matter intake (DMI, kg d^−1^), intake (g kg BW^−1^ d^−1^), time spent eating (min d^−1^) and eating rate (g min^−1^) were all calculated from GrowSafe data for each individual steer on a daily basis. Daily intake variation, measured as coefficient of variation (CV, %), was based on daily intake estimates for individual animals.

To determine time spent lying (min d^−1^), frequency of lying bouts (bouts d^−1^), and duration of lying bouts (min bout^−1^); HOBO accelerometers (HOBO Pendant G acceleration data logger, Onset Corp., Pocasset, MA, USA) were fitted to 12 steers per treatment group. Accelerometers were attached for 15-day increments at the beginning, middle and end of the trial. These devices were programmed to record g-force on the x, y, and z-axes at 1-min intervals and were attached to the front leg above the pastern, as described by Ito et al. [24]. The data loggers were removed from the steers after the 15-day data collection periods and downloaded using Onset HOBO ware software (Onset Corp., Pocasset, MA, USA). From the data, the degree of vertical tilt (*y*-axis) was used to determine whether the animal was lying or standing. Readings <60° indicated that steers were standing, whereas readings ≥60° indicated that steers were lying down.

An Onset HOBO U30-NRC Weather Station (Bourne, MA, USA) was placed near the feedlot and programmed to collect air temperature, relative humidity, wind speed and direction data every 10 min for the entirety of the finishing period. Temperatures adjusted for windchill were calculated using the National Weather Service formula modified for cattle [25,26,27]. Daily average temperatures adjusted for windchill were calculated for the entirety of the finishing period. Short-term relative temperature changes were then derived by subtracting daily average temperatures from a rolling previous 10-day average. Relative temperature change from a rolling 10-day average was used, as previous research results suggest that cattle behavior response to short-term thermal stress was most likely to occur when environmental conditions differ from the 9 to 14-day average [28]. Daily relative temperature change was then paired with daily intake and activity readings for each individual animal for the duration of the finishing period each year. Each day was then classified as colder than the 10-day average (≤−1 SD from the mean), average (±0.5 SD from mean), or warmer than the 10-day average (≥1 SD from the mean) temperature, adjusted for windchill, within each year of the finishing period to evaluate relative temperature change on steer intake behavior (Table S1) and activity (Table S2).

Daily individual intake (kg d^−1^ and g kg BW^−1^ d^−1^), the coefficient of variation of intake, time spent at the feeder, eating rate, and steer lying activity were analyzed using ANOVA (car; [29]) with a generalized linear mixed model (lme4; [30]) including diet, relative temperature change class and the interaction of diet and relative temperature change class as fixed effects, with year and individual steer as random intercepts. Individual steer was used as a random intercept to account for autocorrelation of multiple measurements for each individual. Individual steer was considered the experimental unit. Data were plotted and log transformed if needed to satisfy assumptions of normality and homogeneity of variance. An alpha ≤0.05 was considered significant, and an alpha ≤0.10 was considered a tendency. Orthogonal polynomial contrasts were used to determine linear and quadratic effects of relative temperature change for each analysis, and means were separated using the Tukey method when *p* < 0.05 (emmeans; [31]). All statistical analyses were performed in R [32].

## 3. Results

**Figure 1:**
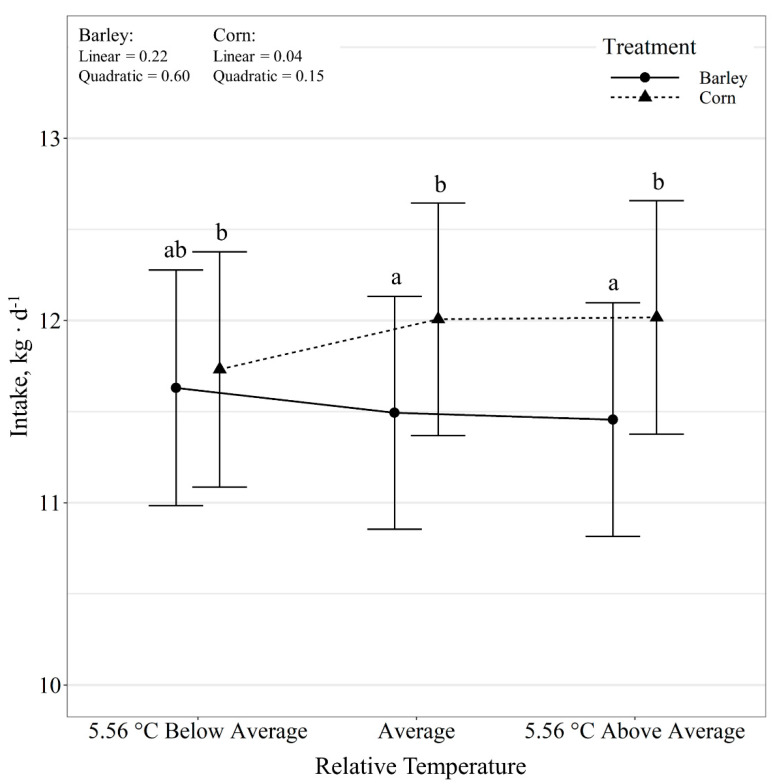
Influence of relative temperature change × diet (*p* = 0.05) on average daily intake (expressed as kg d^−1^) by beef steers consuming either barley or corn-based feedlot diets at the Northern Agricultural Research Center, Havre, MT, USA. Data points without a common letter differ (*p* < 0.05).

**Figure 2:**
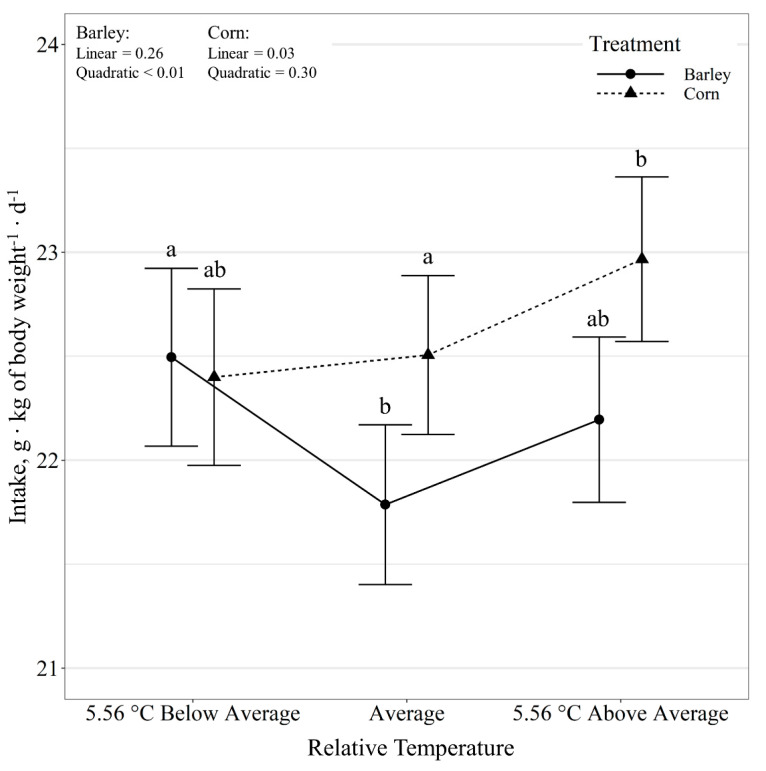
Influence of relative temperature change × diet (*p* = 0.04) on average daily intake (expressed as g kg of body weight^−1^ d^−1^) by beef steers consuming either barley or corn-based feedlot diets at the Northern Agricultural Research Center, Havre, MT, USA. Data points without a common letter differ (*p* < 0.05).

Steer daily intakes displayed a diet by temperature class interaction (*p* = 0.05; Figure 1). Barley-fed steers did not alter daily intakes in response to relative temperature change (*p* ≥ 0.44). However, there was a tendency (*p* = 0.08) for corn-fed steers to alter intake in response to daily temperatures, where intakes decreased linearly (*p* = 0.04) with decreases in relative temperature. Additionally, corn-fed steers had greater (*p* ≤ 0.03) intakes than barley-fed steers on days with average and above average temperature but did not differ (*p* = 0.71) on days below the 10-day average temperature. Steer daily intake expressed as g kg BW^−1^ d^−1^ also displayed a diet by temperature class interaction (*p* = 0.04; Figure 2). There was a quadratic effect (*p* < 0.01) of relative temperature change on intake g kg BW^−1^ d^−1^ for barley-fed steers, where intake increased (*p* = 0.01) on days colder than the 10-day average and tended to increase (*p* = 0.08) on days warmer than the 10-day average. There was a linear effect (*p* = 0.03) of relative temperature change on intake g kg BW^−1^ d^−1^ for corn-fed steers, where intake increased with increases in relative temperature. Diet did not have an effect (*p* = 0.84) on intake g kg BW^−1^ d^−1^ on days colder than the 10-day average; however, corn-fed steers tended (*p* = 0.06) to have greater intakes than barley-fed steers on average and warmer than average days. Relative temperature change had no effect (*p* = 0.60) on variation in intake, expressed as the coefficient of variation. However, diet influenced (*p* < 0.01) the variation in intake, where corn-fed steers had a greater CV than barley-fed steers (21.89 ± 1.46 vs. 18.72 ± 1.46%).

**Figure 3:**
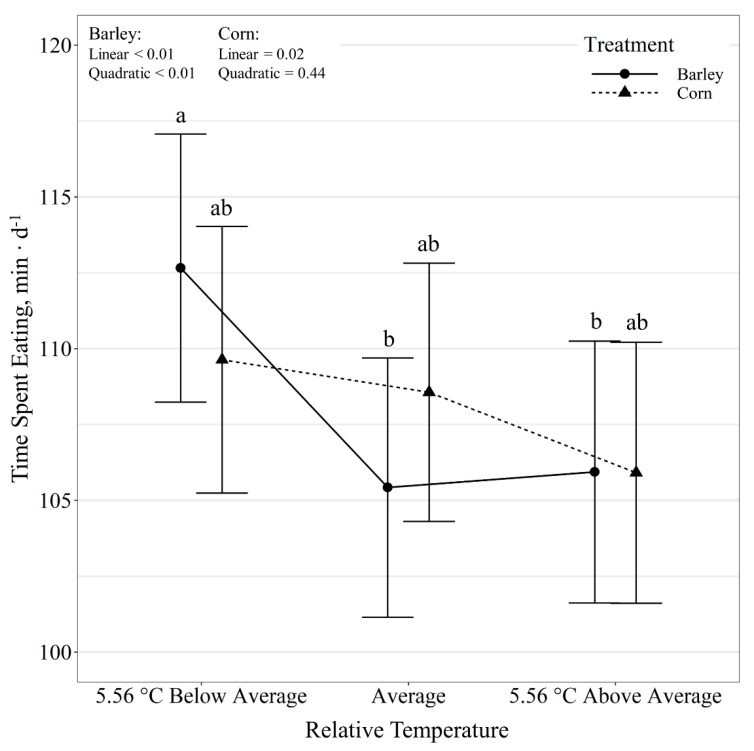
Influence of relative temperature change × diet (*p* < 0.01) on time spent eating (expressed as min d^−1^) by beef steers consuming either barley or corn-based feedlot diets at the Northern Agricultural Research Center, Havre, MT, USA. Data points without a common letter differ (*p* < 0.05).

**Figure 4:**
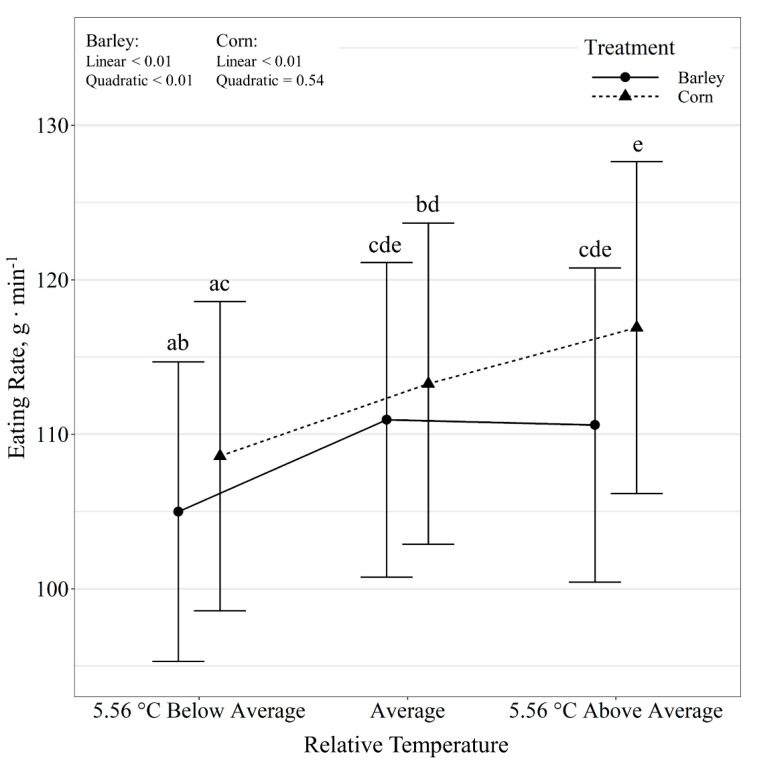
Influence of relative temperature change × diet (*p* = 0.04) on eating rate (expressed as g min^−1^) by beef steers consuming either barley or corn-based feedlot diets at the Northern Agricultural Research Center, Havre, MT, USA. Data points without a common letter differ (*p* < 0.05).

Time spent eating per day also displayed a diet by temperature class interaction (*p* < 0.01; Figure 3). Relative temperature change displayed a quadratic effect of time spent eating per day for barley-fed steers (*p* < 0.01), where time at the feeder increased (*p* < 0.01) on days colder than the 10-day average, however, it did not differ (*p* = 0.89) between days with average and above average temperatures. Corn-fed steers tended (*p* = 0.06) to linearly decrease time at the feeder (*p* = 0.02) as relative temperature increased. Diet had no effect (*p* ≥ 0.40) on time spent eating within each temperature class. Additionally, steer eating rate displayed a diet by temperature class interaction (*p* = 0.04; Figure 4). Relative temperature change displayed a quadratic effect (*p* < 0.01) on eating rate for barley-fed steers, where eating rate decreased (*p* < 0.01) on days colder than the 10-day average, with no differences (*p* = 0.95) observed between days with average and above average temperatures. Corn-fed steers decreased eating rate linearly (*p* < 0.01) with decreasing relative temperatures. Diet had no effect (*p* ≥ 0.33) on eating rate for days with average and below-average temperature, but corn-fed steers tended (*p* = 0.09) to have greater eating rates than barley-fed steers on days warmer than the 10-day average.

**Figure 5:**
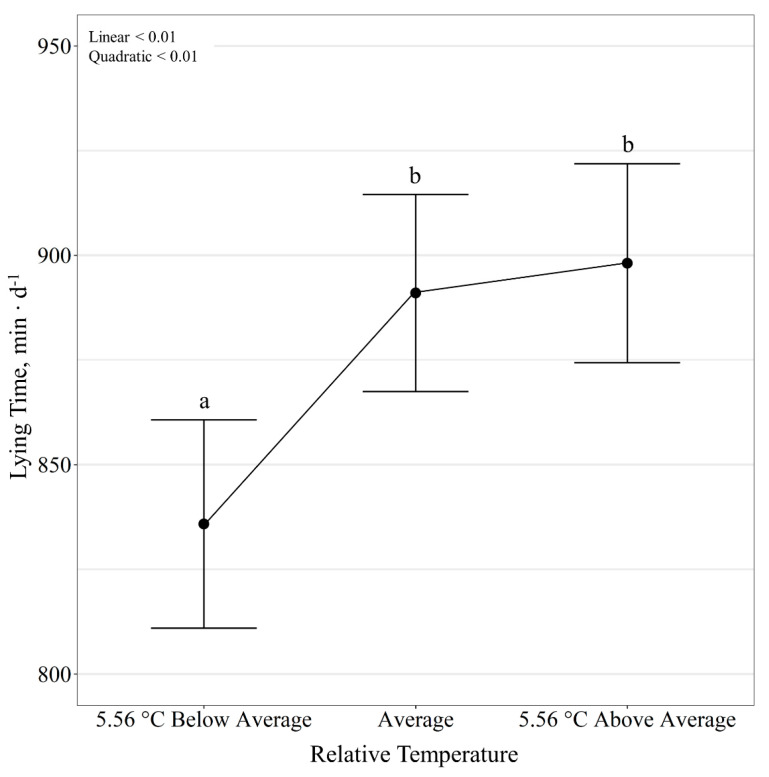
Influence of relative temperature change on lying time (*p* < 0.01; expressed as min d^−1^) by beef steers consuming either barley or corn-based feedlot diets at the Northern Agricultural Research Center, Havre, MT, USA. Data points without a common letter differ (*p* < 0.05).

**Figure 6:**
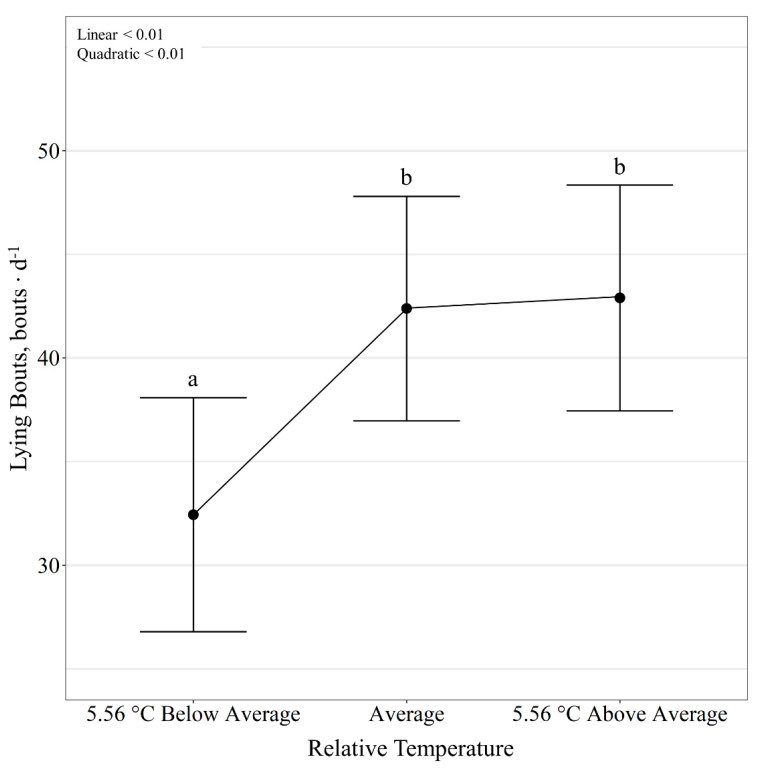
Influence of relative temperature change on lying time (*p* < 0.01; expressed as bouts d^−1^) by beef steers consuming either barley or corn-based feedlot diets at the Northern Agricultural Research Center, Havre, MT, USA. Data points without a common letter differ (*p* < 0.05).

**Figure 7:**
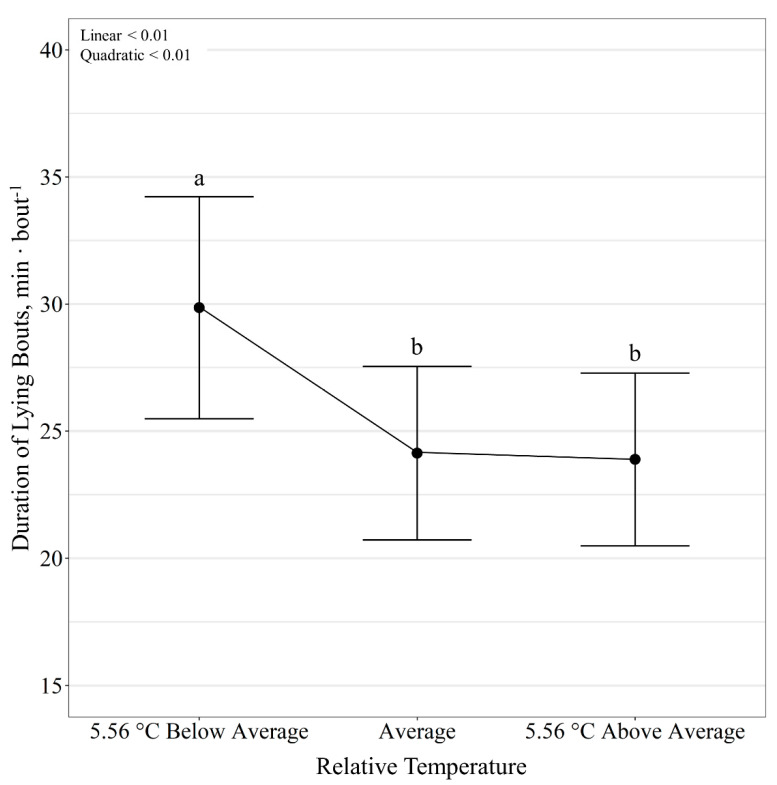
Influence of relative temperature change on lying time (*p* < 0.01; expressed as min bout^−1^) by beef steers consuming either barley or corn-based feedlot diets at the Northern Agricultural Research Center, Havre, MT, USA. Data points without a common letter differ (*p* < 0.05).

Diet had no effect (*p* ≥ 0.12) on steer lying activity; however, temperature class influenced all lying activities (*p* < 0.01). There was a quadratic effect (*p* < 0.01) of relative temperature change on lying time per day, where, regardless of diet, lying time decreased (*p* < 0.01) on days colder than the 10-day average but did not change (*p* = 0.47) between days with average and warmer than the 10-day average temperature (Figure 5). Lying bouts per day was also quadratically influenced (*p* < 0.01) by relative temperature change, where the number of lying bouts decreased (*p* < 0.01) on days colder than the 10-day average but did not change (*p* = 0.90) between days with average and warmer than the 10-day average temperature (Figure 6). Additionally, duration of lying bouts, minutes per day, was quadratically influenced (*p* < 0.01) by relative temperature change, where duration of lying bouts increased (*p* < 0.01) on days colder than the 10-day average temperature but did not differ (*p* = 0.93) between days with average and warmer than the 10-day average temperature (Figure 7).

## 4. Discussion

Variation in beef cattle performance may be attributed to individual feeding behavior and activity [33,34,35]. Changes in environmental conditions have been related to changes in the individual animal’s overall behavior, and environmental shifts are frequently cited as the cause of corresponding alteration in feed consumption, occurrence of ruminal acidosis, and metabolic disorders [36]. In addition, grain sources (corn and barley) can influence feedlot steer behavior [37]. Our results suggest intake expressed on a g kg BW^−1^ d^−1^ basis increased on days colder than the 10-day average for barley-fed steers. However, for corn-fed steers, intake only increased on days above the 10-day average. In grazing beef cattle, mean daily forage intake, expressed as % of BW, increased when temperature deviated (either increase or decrease) from temperature averages [38]. Senft and Rittenhouse [28] concluded that short-term behavioral responses in extreme weather conditions may be critical to the energy balance of domestic animals in both grazing and feedlot scenarios. In feedlot cattle trials, intake increased linearly from 10 °C to −10 °C; however, at temperatures below −10 °C, variation in intake among animals was high, likely due to difference in individual response to cold temperatures [39]. In contrast, our results suggest that relative temperature change did not influence variation in intake. This observation could potentially be due to the uniformity of steers in this study and prior acclimation to the local environment.

It has also been observed that cattle decreased time spent feeding in response to cold temperature [40,41]. Specifically, intake declined in extremely low temperatures because of behavioral patterns, such as standing to shiver, which led to less time eating [39]. Hepola et al. [42] found that low temperatures decreased the time spent eating and growth rate in dairy calves. In our study, time spent eating per day was influenced by temperature change and diet, where barley-fed steers increased time at the feeder on colder than average days, and corn-fed steers tended to linearly increase time at the feeder as temperatures decreased. Interestingly, the eating rate of cattle fed both corn and barley-based diets declined when temperatures were below the 10-day average. While limited work has been conducted on eating rate in relation to environmental changes, increased eating rate has been demonstrated to be associated with increased performance by cattle [43,44], which may aid in explaining the reduction in cattle performance in northern latitudes during winter months [5].

In addition to feed intake, cattle behavioral activity is used as an indication of animal comfort and well-being. Specifically, lying behavior is often a sign of cattle well-being [45,46,47]. Lying and resting behavior are important for cattle, and longer lying times are often associated with better welfare [48]. Periods of rumination are also associated with time spent lying [49]. Conversely, extended lying behavior, >14 h/day, may be a sign of illness, lameness, or disease [50]. Limited work has been conducted specifically evaluating lying response to environmental changes in beef cattle [35]. Tullo et al. [48] recently developed a model that predicted lying behavior in dairy cows based on the temperature–humidity index, solar radiation, air velocity and rainfall. Additionally, dairy cattle have been found to decrease daily lying time, number of lying bouts and lying-bout duration with decreased air temperature [51]. In grazing cattle, shorter lying times have also been reported when cattle experience colder or inclement weather conditions [26]. Our study found that feedlot beef cattle also decreased lying time and lying bouts per day on days colder than the 10-day average. Conversely, other authors have found that cattle exposed to cold temperatures and winter weather conditions increased time spent lying down as temperature decreased; however, cattle in these studies had access to bedding [8,52]. Thus, it has been suggested frozen ground conditions underfoot may impact standing and lying time [51], specifically with a wet or frozen surface contributing a reduction in the time cattle lie down [26,51,53]. Additionally, the conflicting results of the above studies may be due to differences in micro-climates associated with wind and temperature. Increasing wind speed is correlated to convection heat loss, which reduces the temperature an animal experiences [25,27,54]. Therefore, wind can have a profound effect on effective environmental temperature, which could relate to differing animal behavioral responses.

## 5. Conclusions

We found that short-term temperature changes impacted feedlot beef cattle intake behavior and activity. Differing basal diets interacted with short-term environmental changes to influence animal feeding behavior, but diet had limited impact on cattle lying behavior. Overall, environmental shifts and cold temperature conditions could result in greater energetic needs and ultimately impact steer behavior. By providing information related to beef cattle feedlot behavior, we can more effectively manage cattle feeding systems at northern latitudes to improve feed efficiency.

## Data Availability

The data presented in this study are available in Tables S1 and S2.

## Supplementary Materials

https://www.mdpi.com/article/10.3390/ani11051261/s1. Table S1: Animal Feeding Behavior and Weather Data; Table S2: Animal Lying Activity and Weather Data.
